# An empirical study on quality of life and related factors of Pakistani breast cancer survivors

**DOI:** 10.1038/s41598-021-03696-9

**Published:** 2021-12-22

**Authors:** Muhammad Azam, Muhammad Aslam, Javeria Basharat, Muhammad Anwar Mughal, Muhammad Shahid Nadeem, Firoz Anwar

**Affiliations:** 1 Department of Statistics and Computer Science, University of Veterinary and Animal Sciences, Lahore, 54000 Pakistan; 2 Department of Statistics, Faculty of Science, King Abdulaziz University, Jeddah, 21589 Saudi Arabia; 3 Department of Statistics, Kinnaird College for Women, 93-Jail Road, Lahore, 54000 Pakistan; 4 Department of Statistics, Forman Christian College (A Chartered University), Ferozepur Road, Lahore, 54600 Pakistan; 5 Department of Biochemistry, Faculty of Science, King Abdulaziz University, Jeddah, 21589 Saudi Arabia

**Keywords:** Cancer, Diseases, Oncology, Mathematics and computing

## Abstract

A comprehensive understanding of the quality of life (QoL) is essential to establish long-term survivor care plans. The present study was aimed at the assessment of QoL of BC survivors with special emphasis on post-treatment physical, emotional, social, and spiritual challenges. We have assessed the QoL of 250 female BC survivors of all age groups through demographic factors. Volunteer BC survivors were registered in the present study who had got treatment from the Institute of Nuclear Medicine and Oncology (INMOL) hospital and Mayo hospital Lahore. An informed consent form was signed by each participant. The physical, psychological, and spiritual well-being was evaluated by a questionnaire filled with the help of respondents. The average age of BC survivors was 52 ± 7.8 years. Most of them (83%) complained of fatigue during daily life activities, 75.1% body pain or headache, 77.1% had problems with appetite, 63.2% reported weight loss, 77.1% had sleep problems, and 90.5% were feeling general weakness. Only 16.2% were satisfied with their physical health and 2% were not satisfied with their medication. Psychologically, 74.4% were feeling different levels of anxiety, only 10% of them were hoping to achieve a desired life. Age group 21 to 40 years reported better physical health, those with 40–50 years of age and family history of BC have shown better mental strength. The physical and psychological health of survivors from rural areas was comparatively better than those from urban areas. The BC survivor women have to face several physical, psychological and social challenges. The majorities of them complain of anxiety, body pain, fatigue, sleep problems, general weakness, and fear about the future. Our findings suggest the need for psychological support, physical activity a comprehensive post-diagnosis and post-treatment physical and mental health assistance plan for all BC survivors. *Implications for Cancer Survivors*. Survivors of breast cancer experience various challenges including anxiety, sleep problems, body pain, fatigue, and fear about the future. The psychological, physical and social factors make a great difference in their quality of life.

## Introduction

Breast cancer is the most prevalent type of cancer in females and the fifth leading cause of death worldwide with 522,000 cases per year^1^. In developed countries, it is the second leading cause of cancer deaths after lung cancer with 198,000 deaths per year^2^. Breast cancer remains the second leading cause of cancer deaths (246,660 new diagnoses and 40,450 deaths) as projected for 2016 in the USA^3^. In developing countries, breast cancer is the first leading cause of death among women, ranging from 20 to 24.5% of cancer deaths^4^. However, the mortality rate because of breast cancer varies proportionally with several factors including age, stage of diagnosis, the efficiency of treatment procedure, etc.^5^. In developing countries, the etiology of breast cancer is not fully understood and many variations have been found in the tumor behavior, response to treatment, prognosis, and clinical manifestation^6^. Generally, in developed countries, breast cancer is diagnosed at a relatively younger age (60 years) as compared to lung and colorectal cancers which are diagnosed at a median age of 70 and 68 years respectively. According to recent estimates about 19% of breast cancers are diagnosed in women at an age of 30 to 49 years, and 44% are detected in women with an age limit of 65 years or more^7^. For the younger women with age, less than 40 years breast cancer is treated mostly by surgery or mastectomy. Many older women also select these procedures because of fear of recurrence or reluctance to undergo radiation therapy^8,9^. The majority of the women diagnosed with stage I, II, and III undergo mastectomy, and those diagnosed with stage IV are treated with hormone therapy. Because of the improved diagnosis and management procedures, the survival rate of breast cancer has increased 78% to 89% in the USA. Several fundamental physiological changes have been observed among breast cancer survivors including the lack of sensation, tightness in the arms, shoulders, and chest wall, chronic fatigue, and cognitive impairments^10,11^. Treatment with chemotherapy can increase the chances of premature menopause, infertility, osteoporosis^12^ or even neuropathy^13^. Hormonal treatment can improve the risk of atrophic vaginitis and dyspareunia^14^. With an incidence rate of one out of nine women, Pakistan has the highest prevalence of breast cancer in Asia^15^. According to estimates, from December 1995 to December 2009, 45.9% of all diagnosed malignancies among adult women included breast cancer in Pakistan, about 30% of that being diagnosed at stages III or IV^16^. Limited breast health awareness, personal modesty, religious, cultural factors involved in the reluctance to visit a male doctor are the major factors for late diagnosis in Pakistan^17,18^. The present study was aimed at the evaluation of the quality of life (QoL) among the breast cancer survivor women in Pakistan. We have investigated several physicals, physiological, psychological, and behavioral and socio-demographic variables.

Some studies suggested about improvement in the condition of breast cancer survivors and estimated the association of their physical tasks with the health improvement. It has also been concluded that if a survivor did more physical activities at the start of diagnosis, she could easily survive for future life. They proposed that if a breast cancer patient does a long-term physical activity, the possibilities of her continuance with a healthy life increase^19^. Past studies have also discovered a negative relationship between cancer-associated lifestyle preferences, anxiety, and overall well-being. Women attached their breast cancer to lifestyle-related problems and are recognized to practice suffering that their activities could have added to the spread of their sickness^20,21^. Spirituality is the dimension of life that allows an individual to interact and unite with the world. Interaction and assimilation give purpose and worth to human life and promote it. Moral/spiritual gathering therapy is a kind of psychotherapy that brings against particular policies and spiritual methods to permit subjects to achieve a nonmaterial perception of themselves, world, events, appearances, and finally well-being and completion^22^. Another study showed that sufferers should be reassured to endeavor spirituality to dispense with physical and psychological rejoinders to cancer effectively^23^. More inadequate social channels and understandings of insufficient social support may badly influence psychological adjustment^24^ and endurance^25,26^. Poor social interfaces^27^, weak social maintenance^28^, and low social well-being^29^ are affiliated with a heightened death rate in breast cancer.

By exploring the literature and according to the best of the author’s knowledge, there is the least work on studying the factors that cause breast cancer in Pakistani women. Moreover, factors of life quality of BC survivors studied previously, do not fit in the Pakistani context due to differences in several areas such as living styles, status of families, social, cultural, and religious values, healthcare facilities variations, etc. For this study, the questionnaire established by the City of Hope National Medical Center has been mainly considered. The instrument developed by CHNMC measures the quality of life of BC survivors wherein the components of life that link the human being with the society reflect the QoL. Also, in the said instrument, demographic variables are not included which can play a significant role in one’s life quality particularly in the Pakistani context. The instrument for this study has been designed after interviewing a number of patients of various hospitals and experts dealing with BC patients. The instrument consists of few demographic variables other than constructs which represent general life quality and four factors measuring patients’ well-being. Items of constructs have been designed specifically that fit best in the Pakistani context so far as social, cultural, and religious values are concerned and they statistically fulfill the reliability and validity criteria. This study is mainly focused on assessing QoL with other dimensions of breast cancer survivors who come across their lives and how these factors regress the QoL. This study also discusses the significance of demographics within measurements and their impact on QoL.

## Materials and methods

### Study design, recruitment, and sampling

The QoL Breast Cancer instrument is based on prior versions of the QoL instrument by researchers at the City of Hope National Medical Center. The updated tool encompasses 46 items depicting the four quality of life domains, including physical, psychological, social, and spiritual well-being. This study was administrated as a face-to-face survey at INMOL hospital, Lahore. Psychometric analysis was done on 250 female BC survivors as respondents, including tests of reliability and validity. The overall QoL-CS tool test reliability was 0.88 with subscales of physical 0.89, psychological 0.85, social 0.82, spiritual 0.80. The measure of reliability was the estimation of internal consistency employing Cronbach's alpha coefficient. Analysis exhibited an overall 0.95. Subscale alphas ranged from 0.75 for spiritual well-being, 0.78 for physical, 0.89 for social, and 0.84 for psychological. Reliability and validity tests were employed to ascertain the degree to which the instrument estimates the QoL and other factors in cancer survivors. The measure of validity was tested by Pearson’s correlation between items and the total of construct’s items. To determine the relation of subscales of the QoL-CS, multiple regression and Pearson's correlations are in the application.

This cross-sectional and analytical study was conducted on the breast cancer survivors after the completion of their treatment. The women of age up to 80 years, who had got treatment at least 1 year before and did not experience relapse were included in the study. The women who have been in hospital because of some other disease and were severely ill were excluded from the study. The study was conducted with the breast cancer patients who had completed their treatment from INMOL Hospital, Mayo Hospital Lahore have been assessed for quality of life (QoL) variables. The majority of the patients had been associated with INMOL hospital, mostly from the same socio-economic group. A total of 250 patients were interviewed by female authors in 12-month duration, from January 2020 to December 2020. In Pakistani culture and society, it is hard for the male surveyors to interview women in the BC context. A questionnaire consisting of different physical, physiological, psychological, behavioral, and socio-demographic QoL variables was filled by each patient or by the information provided by the patient. Each patient was provided with complete information about the aims and objectives of the present study. SPSS software with version 17.0 has been used to analyze the results. The ethical research committee from Kinnaird College approved the study. We confirm that all methods were performed in accordance with the relevant guidelines and regulations.

### Statistical analysis of data

Regression analysis was applied to analyze the average relationship among two or more variables. Simple linear regression is a relationship that elaborates the dependency of response variables for the given value of the independent variable.

### Ethical approval

This article does not contain any studies with animals performed by any of the authors.

### Informed consent

Informed consent was obtained from all individual participants included in the study.

### Ethical committee approval

The ethical committee approved this research under the reference number KC/2019-2/PKEDU.

## Results

### Socio demographic variables

**Figure 1:**
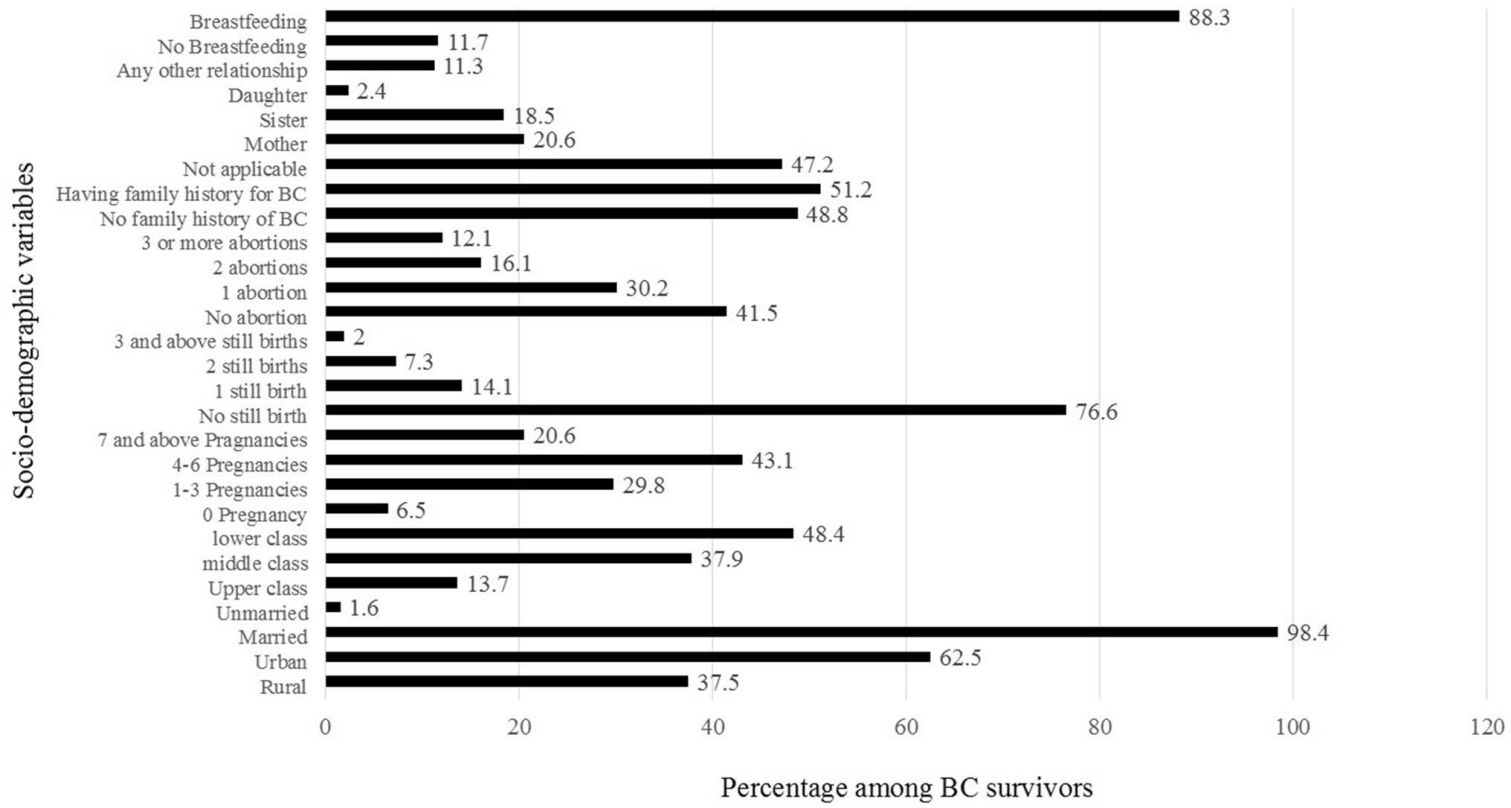
Percentage occurrence of different socio-demographic QoL variable among the BC survivors.

Total of 250 breast cancer survivors were registered evaluated for several life quality variables. The average age of the patients was 52 ± 7.8 years, all were females, 245 (98%) of them were married and 5 (2%) were unmarried. About 78% of survivors had gone through lumpectomy or mastectomy, 14% radiation therapy, and 8% had received chemotherapy. An assessment for socio-demographic variables includes; residential area, social status, marital status, post- treatment pregnancy, stillbirth and abortion, family history of breast cancer, breastfeeding, or non-breastfeeding (Fig. 1).

### Physiological, psychological and spiritual well-being of survivors

**Figure 2:**
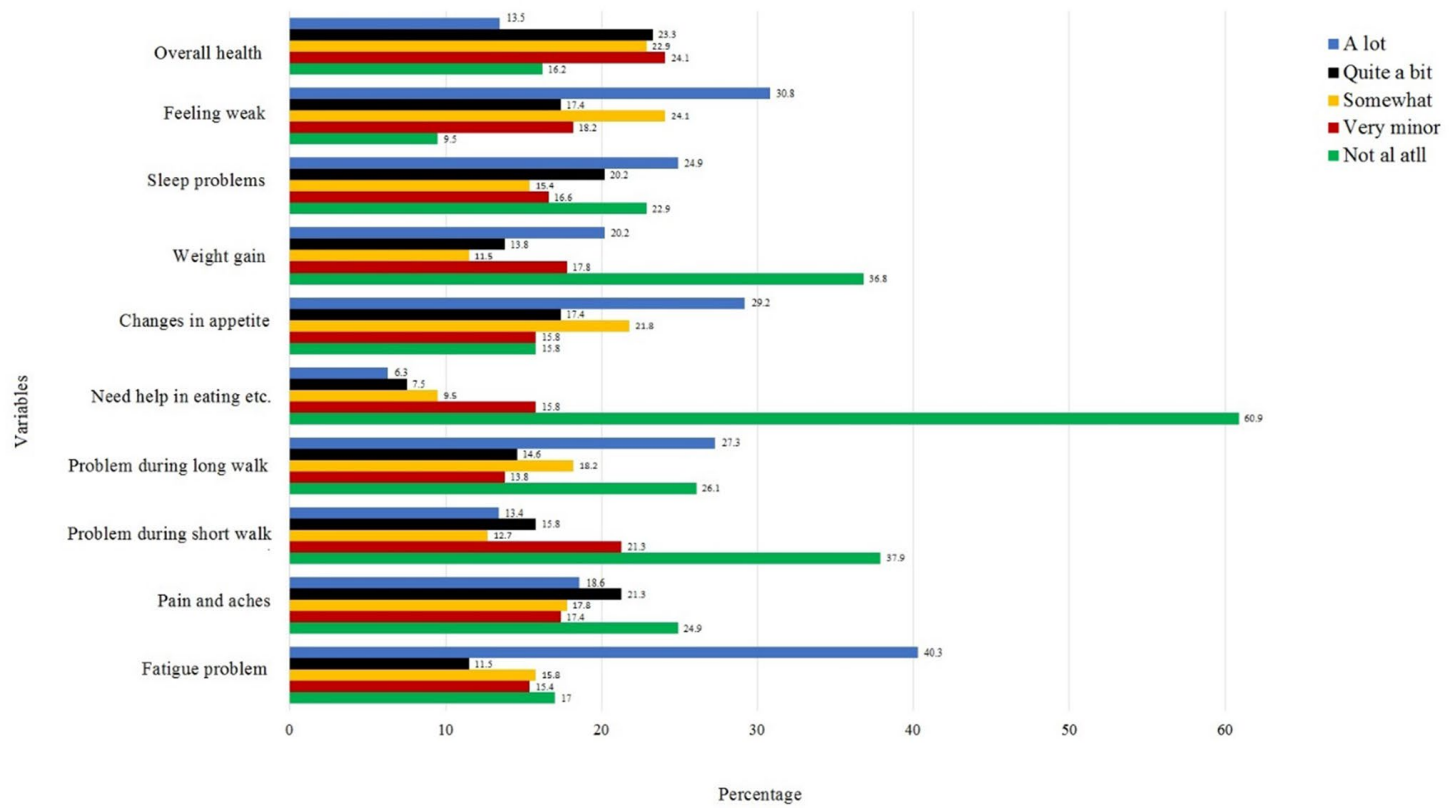
Variables of QoL and physical well-being among the BC survivors.

**Figure 3:**
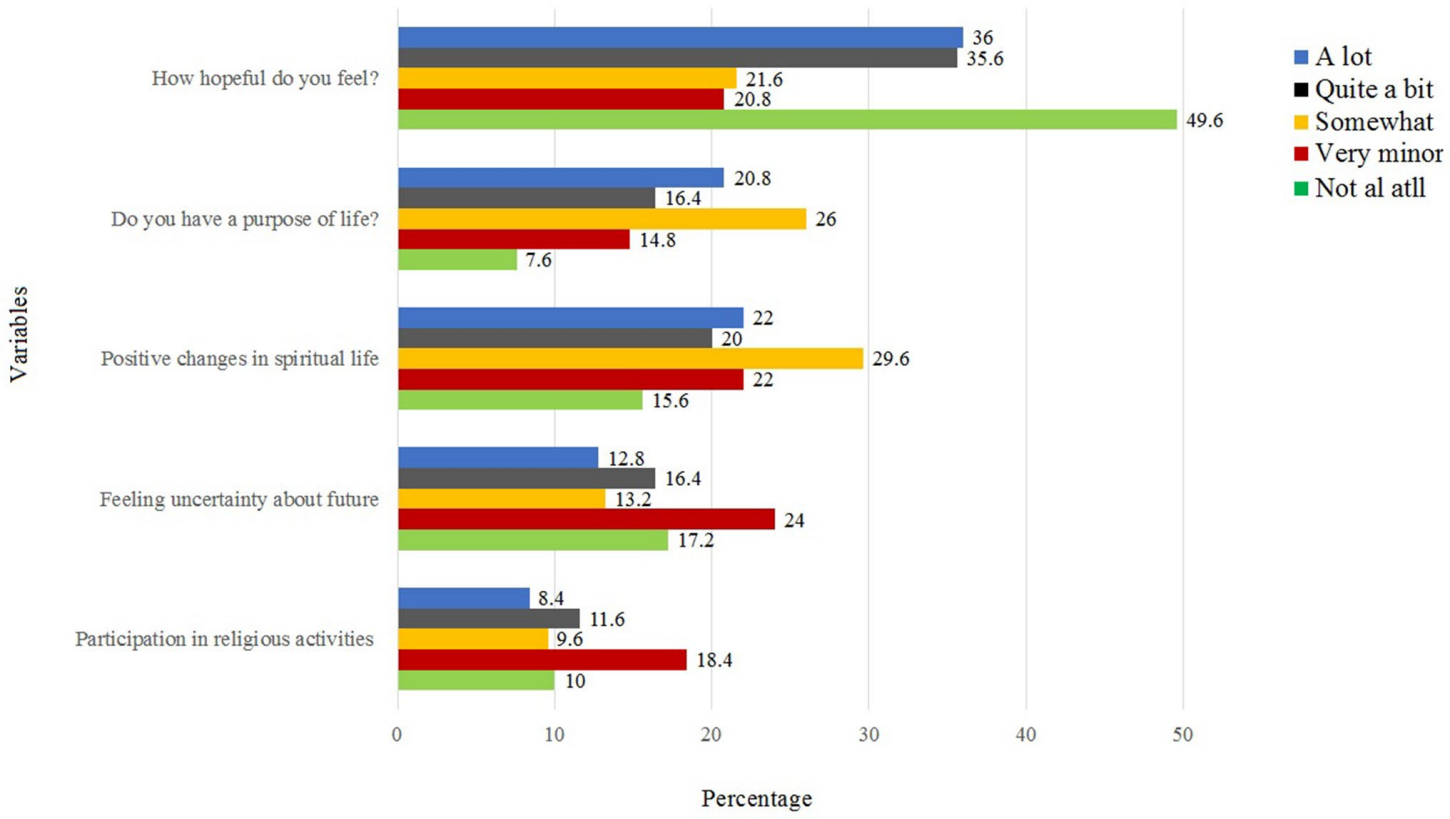
Variables for mental health assessment among the BC survivors.

**Figure 4:**
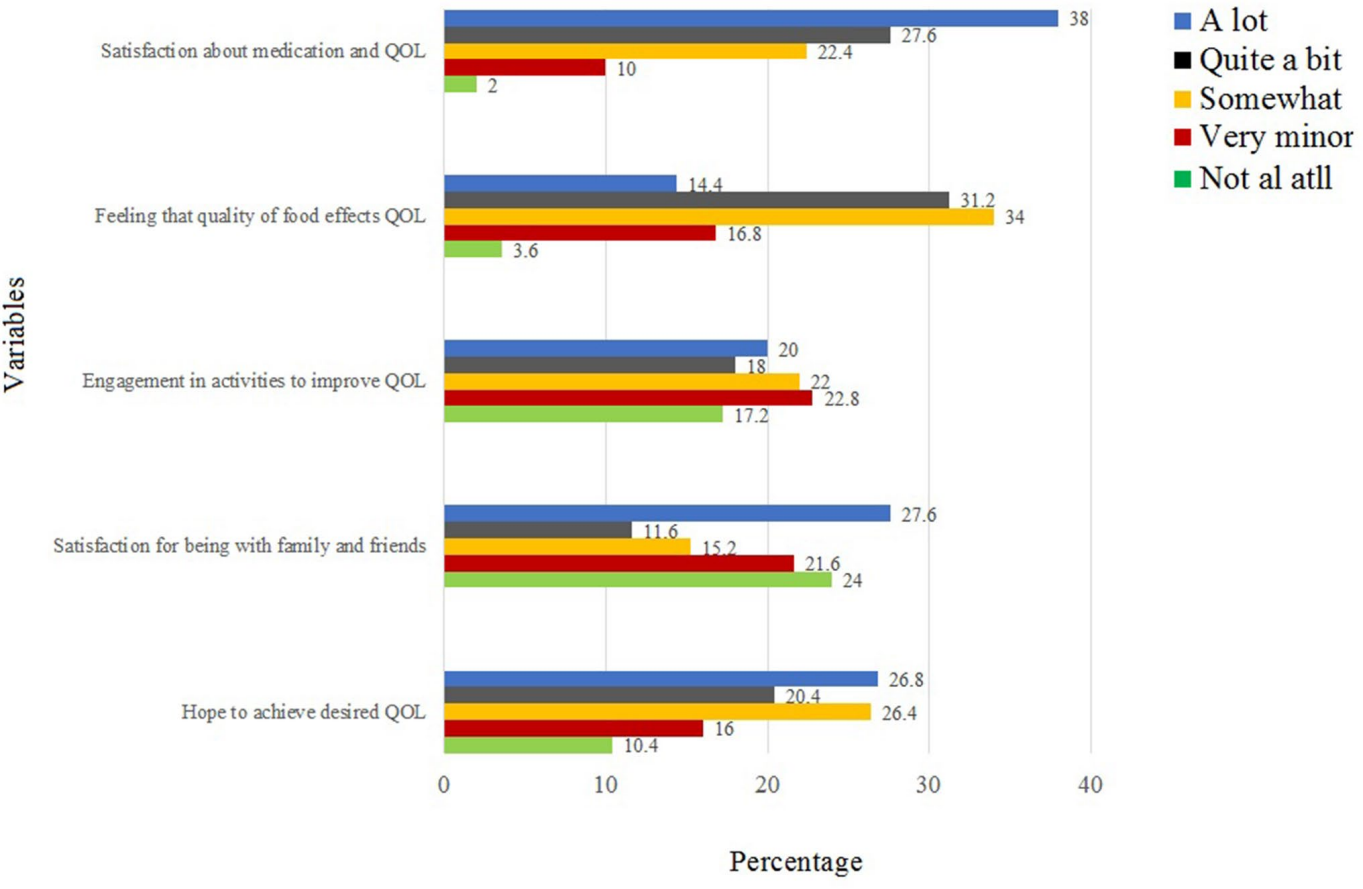
Variables for the analysis of spiritual and religious well-being of BC survivors.

The majority (83%) of BC survivors were feeling variable degrees of fatigue during the performance of their daily life activities. Severe to mild body pain and headache were reported among 75.1% of participants. Generally, there was not much problem during a short walk but 73.9% had difficulties in a long walk. Issues with appetite were reported by 77.1%, loss of weight by 63.2%, sleep problems by 77.1%, and general weakness by 90.5% of participants. Only 16.2% of BC survivors have shown satisfaction with their physical health (Fig. 2). Psychologically, 86.8% reported distress after initial diagnosis, during chemotherapy (85.6%), radiation therapy (73.2%), and surgery (74.8%). A majority (74.4%) of participants was feeling different level of anxiety and depression after recovery and 73.2% were afraid of a recurrence of cancer in the future (Fig. 3). Overall, the BC survivors were very hopeful about their health, satisfied while spending time with their family and friends. The majority of them were involved in religious activities and they seem to be hopeful about their future (Fig. 4). Only 2% of survivors were not satisfied with their medication facilities, the majority of them had a view that quality of food matters in the improvement of their health. Only 10% of survivors were hopeful to achieve their desired quality of life.

### Life quality on the basis of age, marital and social status

With the mean value of 2.9 ± 0.58, BC survivors of 21 to 40 years of age have better physical health. The psychological health of 41 to 50-year-old participants was better with the highest mean value of 3.25 ± 0.7. Survivors up to 40 years of age have strong tendencies towards spiritual and religious activities. Overall, the 60 plus age group had the lowest life activities. There was no significant effect of the marriage age of patients on their physical, psychological recovery, and religious trends (Table 1). On the basis of mean values, we found that the physical and psychological health of survivors from rural areas was comparatively better than those from urban areas. However, the respondents from urban areas enjoy a better quality of life and have more tendencies towards spiritual and religious activities. However, no significant effect of marital status was recorded on the life quality variables (Table 1).

**Table 1:** Descriptive analysis of psychological, physical, spiritual and overall health status of breast cancer survivors by dividing into various categories.

Life quality variables	Patient age category					Marriage age category				
	Patient age	N	Mean	S.D	S.E	Marriage age	N	Mean	S.D	S.E
Physical wellbeing	Up to 20	2	1.60	0.283	0.200	Up to 14	13	3.45	0.532	0.147
	21–30	18	2.98	0.585	0.138	15–19	71	3.00	0.693	0.082
	31–40	50	2.90	0.581	0.082	20–24	105	2.79	0.575	0.056
	41–50	64	2.78	0.638	0.080	25–29	44	2.82	0.514	0.077
	51–60	56	2.76	0.564	0.075	30 plus	12	3.05	0.601	0.173
	61–80	60	3.17	0.620	0.080	–	–	–	–	–
	Total	250	2.90	0.629	0.040	Total	245	2.90	0.618	0.039
Psychological wellbeing	Up to 20	2	2.44	0.619	0.438	Up to 14	13	3.01	0.939	0.260
	21–30	18	3.03	0.833	0.196	15–19	71	3.13	0.784	0.093
	31–40	50	2.97	0.768	0.109	20–24	105	3.13	0.735	0.072
	41–50	64	3.25	0.703	0.088	25–29	44	3.00	0.765	0.115
	51–60	56	3.11	0.707	0.094	30 plus	12	2.86	0.870	0.251
	61–80	60	3.05	0.873	0.113	–	–	–	–	–
	Total	250	3.09	0.771	0.049	Total	245	3.09	0.770	0.049
Spiritual wellbeing	Up to 20	2	3.90	0.141	0.100	Up to 14	13	2.69	0.922	0.256
	21–30	18	3.72	0.606	0.143	15–19	71	3.24	0.683	0.081
	31–40	50	3.68	0.716	0.101	20–24	105	3.56	0.639	0.062
	41–50	64	3.38	0.605	0.076	25–29	44	3.57	0.603	0.091
	51–60	56	3.46	0.599	0.080	30 plus	12	3.53	0.657	0.190
	61–80	60	3.13	0.775	0.100	–	–	–	–	–
	Total	250	3.43	0.695	0.044	Total	245	3.42	0.696	0.044
Overall quality of life	Up to 20	2	4.00	0.849	0.600	Up to 14	13	2.45	0.984	0.273
	21–30	18	3.83	0.786	0.185	15–19	71	3.22	0.987	0.117
	31–40	50	3.64	0.941	0.133	20–24	105	3.42	0.831	0.081
	41–50	64	3.52	0.801	0.100	25–29	44	3.50	0.937	0.141
	51–60	56	3.21	0.758	0.101	30 plus	12	3.32	0.674	0.195
	61–80	60	2.78	0.958	0.124	–	–	–	–	–
	Total	250	3.32	0.924	0.058	Total	245	3.32	0.922	0.059

	Family history of breast cancer					Breast feeding category				
	F H	N	Mean	S.D.	S.E.	B F	N	Mean	S.D.	S.E.
Physical wellbeing	No	123	2.99	0.606	0.055	No	30	2.84	0.752	0.137
	Yes	127	2.81	0.639	0.057	Yes	219	2.91	0.613	0.041
	Total	250	2.90	0.629	0.040	Total	249	2.90	0.630	0.040
Psychological wellbeing	No	123	3.05	0.837	0.076	No	30	3.02	0.655	0.120
	Yes	127	3.13	0.702	0.062	Yes	219	3.10	0.787	0.053
	Total	250	3.09	0.771	0.049	Total	249	3.09	0.771	0.049
Spiritual wellbeing	No	123	3.57	0.690	0.062	No	30	3.41	0.678	0.124
	Yes	127	3.29	0.673	0.060	Yes	219	3.43	0.700	0.047
	Total	250	3.43	0.695	0.044	Total	249	3.43	0.696	0.044
Overall quality of life	No	123	3.45	0.958	0.086	No	30	3.53	0.918	0.168
	Yes	127	3.20	0.874	0.078	Yes	219	3.30	0.922	0.062
	Total	250	3.32	0.924	0.058	Total	249	3.33	0.923	0.058

	Abortion category					Still birth category				
	Abortion	N	Mean	S.D.	S.E.	Still birth	N	Mean	S.D.	S.E.
Physical wellbeing	No abortion	104	2.95	0.602	0.059	No still birth	190	2.92	0.596	0.043
	1 abortion	75	2.83	0.622	0.072	1 still birth	35	2.76	0.733	0.124
	2 abortions	40	2.81	0.625	0.099	2 still births	18	2.91	0.697	0.164
	3 and above	30	3.05	0.680	0.124	3 and above	6	3.23	0.468	0.191
	Total	249	2.90	0.623	0.039	Total	249	2.90	0.623	0.039
Psychological wellbeing	No abortion	104	3.04	0.757	0.074	No still birth	190	3.10	0.755	0.055
	1 abortion	75	3.15	0.796	0.092	1 still birth	35	3.17	0.758	0.128
	2 abortions	40	3.03	0.752	0.119	2 still births	18	3.13	0.848	0.200
	3 and above	30	3.22	0.780	0.142	3 and above	6	2.35	0.871	0.356
	Total	249	3.09	0.769	0.049	Total	249	3.09	0.769	0.049
Spiritual wellbeing	No abortion	104	3.60	0.644	0.063	No still birth	190	3.46	0.714	0.052
	1 abortion	75	3.40	0.677	0.078	1 still birth	35	3.29	0.626	0.106
	2 abortions	40	3.29	0.634	0.100	2 still births	18	3.24	0.660	0.156
	3 and above	30	3.05	0.820	0.150	3 and above	6	3.73	0.450	0.184
	Total	249	3.42	0.696	0.044	Total	249	3.42	0.696	0.044
Overall quality of life	No abortion	104	3.49	0.895	0.088	No still birth	190	3.32	0.934	0.068
	1 abortion	75	3.39	0.818	0.094	1 still birth	35	3.22	0.852	0.144
	2 abortions	40	3.09	0.931	0.147	2 still births	18	3.28	0.921	0.217
	3 and above	30	2.83	1.060	0.193	3 and above	6	3.80	1.020	0.416
	Total	249	3.32	0.922	0.058	Total	249	3.32	0.922	0.058

	Rural/urban category					Married/unmarried status				
	Area	N	Mean	S.D.	S.E.	Status	N	Mean	S.D.	S.E.
Physical wellbeing	Rural	94	3.08	0.614	0.063	Married	245	2.90	0.618	0.039
	Urban	156	2.79	0.615	0.049	Unmarried	5	2.74	1.122	0.502
	Total	250	2.90	0.629	0.040	Total	250	2.90	0.629	0.040
Psychological wellbeing	Rural	94	3.24	0.715	0.074	Married	245	3.09	0.770	0.049
	Urban	156	3.00	0.791	0.063	Unmarried	5	3.13	0.901	0.403
	Total	250	3.09	0.771	0.049	Total	250	3.09	0.771	0.049
Spiritual wellbeing	Rural	94	3.16	0.712	0.073	Married	245	3.42	0.696	0.044
	Urban	156	3.59	0.635	0.051	Unmarried	5	3.68	0.672	0.301
	Total	250	3.43	0.695	0.044	Total	250	3.43	0.695	0.044
Overall quality of life	Rural	94	2.93	0.924	0.095	Married	245	3.32	0.922	0.059
	Urban	156	3.56	0.843	0.068	Unmarried	5	3.36	1.117	0.500
	Total	250	3.32	0.924	0.058	Total	250	3.32	0.924	0.058

### History of abortion, stillbirth breastfeeding and family history categories

The respondents with no case of abortion had comparatively better physical health as indicated by a higher mean value of 2.95 ± 0.5 and low standard deviation. No significant difference was found in psychological health. However, the survivors with no abortion cases became more religious and more determined to acquire a better life. No significant difference could be detected from the data provided by survivors having stillbirth cases (Table 1). No significant difference was determined in the physical health of survivors with and without a family history of breast cancer. However, those with a family history of BC were psychologically better with a mean value of 3.13 ± 0.7. The physical and psychological health of survivors having breastfeeding history was significantly better than those having no breastfeeding (Table 1). The majority of 120 (48%) of BC survivors were from a lower class, 95 (38%) were from the middle class, and 35 (14%) were from the upper social or economic class. The mean value for the physical well-being of the upper class was 3.11 ± 0.7, for middle class 2.98 ± 0.6, and for the lower class it was 2.50 ± 0.7. Similarly, the psychological, spiritual well-being, and overall quality of life of upper-class survivors were better than those of the lower class (Table 1).

### Correlations between QoL, physical wellbeing, psychological well-being, and spiritual well being

There is a significant negative low relationship exists between physical well-being and quality of life. It determines if the physical well-being of patients decreases, then their quality of life will improve (Table 2). There is a significant negative low relationship exists between psychological well-being and quality of life. It indicates if the psychological well-being of patients decreases, then their quality of life will improve (Table 3). Also, there is a significant positive moderate relationship exist between spiritual well-being and quality of life. It means if the spiritual well-being of patients increases, their quality of life will also increase moderately (Table 4). It has also been observed a significant positive moderate relationship between social well-being a quality of life. This means that being connected socially can give a rise to your mental health and improve your quality of life (Table 4).

**Table 2:** Correlation between physical well-being and QoL.

	1	2	3	4
**1. QoL**				
Pearson correlation	1	− .340	− .321	.542
Sig. (2-tailed)		.000	.000	.000
N	250	250	250	250
**2. Physical well being**				
Pearson correlation	− .340	1	.231	− .118
Sig. (2-tailed)	.000		.000	.062
N	250	250	250	250

**Table 3:** Correlation between psychological well-being and QoL.

	1	2	3	4
**1. QoL**				
Pearson correlation	1	− .340	− .321	.542
Sig. (2-tailed)		.000	.000	.000
N	250	250	250	250
**2. Psycho well being**				
Pearson correlation	− .321	.231	1	− .194
Sig. (2-tailed)	.000	.000		.002
N	250	250	250	250

**Table 4:** Spiritual well-being and QoL.

	1	2	3	4
**1. QoL**				
Pearson correlation	1	− .340	− .321	.542
Sig. (2-tailed)		.000	.000	.000
N	250	250	250	250
**2. Spiritual well being**				
Pearson correlation	.542	− .118	− .194	1
Sig. (2-tailed)	.000	.062	.002	
N	250	250	250	250

### Regression analysis

There is a statistically significant effect of Physical Well-being, Psychological well-being, spiritual well-being, and Social Well-Being on QoL of breast cancer patients at a 5% level of significance., but Social Well-being (SWB) has not a significant effect on the QoL of breast cancer survivors. We can conclude that the physical exertion, psychological well-being, and spiritual well-being of breast cancer survivors have a positive impact on their QoL (Table 5). The regression weights of independent variables are shown in Table 6.

**Table 5:** Social well-being and QoL.

	1	2	3	4
**1. QoL**				
Pearson correlation	1	− .124	− .231	.653
Sig. (2-tailed)		.000	.000	.000
N	250	250	250	250
**2. Social well being**				
Pearson correlation	.653	− .259	− .148	1
Sig. (2-tailed)	.000	.032	.001	
N	250	250	250	250

**Table 6:** Regression weights of independent variables.

Model	Unstandardized coefficients		Standardized coefficients	t	Sig
	B	Std. error	Beta		
(Constant)	3.803	.703		5.413	.000
Physical well-being	− .124	.058	− .121	− 2.158	.032
Psychological well-being	.498	.068	.450	7.366	.000
Spiritual well-being	− .069	.048	− .072	− 1.414	.001
Social well-being	.121	.054	.125	2.244	.026

## Discussion

Breast cancer, associated with many physiological, genetic, lifestyle and environmental factors has the highest prevalence in women and represents the leading cause of cancer-related deaths among women worldwide^1–4^. According to recent estimates, the BC has a highly variable incidence rate in different geographical areas with significantly higher prevalence in the developed countries as compared to developing countries. The disease has a continuously increasing health burden worldwide^4^. With the availability of early diagnostic tools and treatment procedures, the survival rate of breast cancer patients has rapidly increased in recent years. According to the recent data, more than a 31% increase in the BC survivors has been observed during the last two decades^5^. The rapidly increased survival rate has emphasized the need to maintain the quality of life and longevity of BC survivors. In this context, the present study was aimed at the collection of data for physiological, social, and psychological problems faced by the post BC treatment survivors. The present study involves 250 BC survivors after at least 1 year of their treatment. A big majority of survivors (98%) were married, 2% were unmarried and their average age was 52 ± 7.8 years at diagnosis. About 78% of survivors had gone through lumpectomy or mastectomy, 14% radiation therapy, and 8% had received chemotherapy. None of the survivors was treated with conventional herbal medicine etc. We have analyzed the physical, psychological, social, and spiritual well-being and QoL of survivors. In general, the survivors included in the present study have reported poor quality of life. According to the present study, 83% of BC survivors experienced mild to severe fatigue, 75.1% reported headache or body pain. If we compare with the literature available, the maximum fatigue rate recently reported in prostate cancer survivors has been 66.9%^19^, among lung cancer survivors it has been 74%^20^. Overall, 50% to 90% of survivors have been reported to complain of fatigue^21^. More than 80% of oncologists believe that fatigue can interfere with the daily life activities of cancer survivors and medical staff should consider the possibility of fatigue among survivors^20^. The body pain among BC survivors has been reported in the literature with reference to its association with the surgery type and psychological factors^22^, post-treatment depression has clearly been associated with pain^23^. Most of the survivors included in our investigation had no major issue in a short walks, however, 73.9% of them were having mild to a serious problem in long walks indicating a lack of physical activity. According to more than 20 research reports exercise has been a positively supportive intervention on the recovery of breast cancer survivors^24,25^. Exercise also has some positive impact on cardiorespiratory fitness; it reduces fatigue, anxiety, and depression^26^. The loss of appetite, body weight, and weakness has been a general trend in the BC survivors included in the present study. According to the literature, obesity is clearly established risk factor for many types of cancer^27^, weight gain after a cancer diagnosis can increase the risk of mortality significantly^28^. Hence, weight loss is a positive factor for the life quality of BC survivors^29,30^. However, the loss of appetite and general weakness will disease the overall QoL of survivors. Survivors, who reported level the highest level of distress and anxiety at diagnosis, at chemotherapy, radiation therapy, and surgery. The level of anxiety was reduced after treatment. However, 73.2% were afraid of disease recurrence. Previously, higher levels of distress have been reported among females during chemotherapy or radiation therapy^31,32^. The level of anxiety diseases after the completion of treatment but remained elevated if the survivors have less social interaction^33^. To overcome post-treatment anxiety, the cancer survivors should be supported by health professionals, psychologist, relatives, and society^34,35^. In our case, only 10% of respondents were optimistic to achieve the desired quality of life indicating a severe need for social, psychological, and physical assistance for the survivors. In the present study, an assessment of QoL variables was made on the basis of age, marital and social status of survivors. The age group 21 to 50 years have shown better physical and psychological health respectively. The survivors of 60 years have shown the lowest life activities. We found no effect of marriage age on survivors (Table 1). Physical and psychological improvement of patients from rural areas was generally better. However, the survivors from urban areas have better QoL and more tendency towards religion. Our findings are similar to many reports indicating slightly better psychological health of old age survivors^36–39^. However, our results are not in accordance with the findings suggesting that below 20 years full-time pregnancy protects or reduces the chances of breast cancer^40^. The respondents with no abortion case post-treatment had better physical health. However, no significant difference was found in psychological health on the basis of abortion history and stillbirth history. We established that the better physical health of BC survivors could be a relative health weakening contributed by abortion or stillbirth. We found no significant difference in the physical health of cancer survivors on the basis of family history. The psychological health of those with a family history of BC was relatively better (Table 1). The physical and physiological health of survivors with breastfeeding history was comparatively better. Our results support the previous findings that breastfeeding may reduce the chances of cancer recurrence up to 30% by the establishment of an anti-proliferative environment in the tumor^41^. In the present study, we also found a positive relationship between social status and the overall well-being of survivors. Studies during last the two decades have suggested that the social milieu of patients and survivors makes a big difference^42–44^. Generally, the mortality rate is doubled among females with pre-diagnosis social isolation^44^. Better social status and social support improve the physical and mental QoL^45–54^.

## Conclusion

Survivors of breast cancer experience various challenges including anxiety, sleep problems, body pain, fatigue, and fear about the future. The psychological, physical, and social factors make a great difference in their quality of life. Our findings emphasize the requirement of a comprehensive post-treatment physical and mental health assistance plan for all BC survivors. The BC survivors must be approached by a team of medical experts with multidisciplinary training, integrating individually on the bunch of post-treatment problems faced by the patients. Written information can be provided to the survivors and training sessions can be arranged by the hospitals and healthcare authorities. The caretakers and family members should be properly informed about the importance of social support. Both clinical and psychological settings can be provided to improve the life quality and longevity of BC survivors. The information provided in the present study can be prolonged to establish the surveillance mechanisms by the hospitals and healthcare authorities to improve the QoL issues among BC survivors.

## Data Availability

All primary data and further information required in the future is available with first author and corresponding author.
